# Ultra-deep sequencing of HIV-1 near full-length and partial proviral genomes among chronically infected blood donors at four blood centers in Brazil

**DOI:** 10.1186/1471-2334-14-S2-P61

**Published:** 2014-05-23

**Authors:** Rodrigo Pessôa, Jaqueline Tomoko Watanabe, Paula Loureiro, Maria Esther Lopes, Anna Barbara Carneiro-Proietti, Ester C Sabin, Michael P Busc, Sabri S Sanabani

**Affiliations:** 1 Institute of Tropical Medicine, University of São Paulo, São Paulo, Brazil; 2 Pernambuco Hematology and Hemotherapy Institute - HEMOPE, Recife, PE, Brazil; 3 Rio de Janeiro Hematology and Hemotherapy Institute - HEMORIO, Rio de Janeiro, Brazil; 4 Federal University of Minas Gerais - UFMG, Belo Horizonte, MG, Brazil; 5 Department of Infectious Diseases, University of São Paulo, São Paulo, Brazil; 6 Blood Systems Research Institute, San Francisco, California 94117, USA

## Introduction

Here, we aimed to gain a comprehensive picture of HIV-1 diversity in the north-east and south-east part of Brazil. To this end, a high-throughput sequencing was used to characterize the near full length (NFLG) and partial HIV-1 proviral genome in blood donors at four major blood centers in Brazil: Pro-Sangue foundation (São Paulo state (SP), n 48), Hemominas foundation (Minas Gerais state (MG), n 41), Hemope foundation (Recife state (PE), n 97) and Hemorio blood bank (Rio de Janeiro (RJ), n 90).

## Material and methods

From 2007-2011, 341 HIV+ blood donors from 4 blood centers were recruited to participate in a case control study to identify risk exposure. Of those, 294 specimens were classified as chronically infected subjects based on reactivity and positivity on the tests for recent HIV infection. Five overlapping amplicons spanning the HIV genome were PCR amplified from PBMCs. The amplicons were molecularly bar-coded, pooled, and sequenced by Illumina protocol.

## Results

Of the 294 samples studied, 229 (77.9%) NFLGs and 50 (17%) partial fragments were de novo assembled into contiguous sequences and successfully subtyped. Of those, 200 (71.7%) were pure subtypes consisting of clade B (n = 151, 75.5%), C (n = 11, 5.5%) F1 (n = 4, 2%) and D (n = 3, 1.5%). Recombinant viruses were detected in 79 (28.3%) samples and consist of BF1 (n = 67, 84.8%), BC (n = 6, 7.6%), BCF1 (n = 4, 5%), CF1 and BUA1 (n = 1, 1.2%, each). Evidence of dual infection with the same subtype was detected in 1 patient. Two distinct BF1 recombinant profiles based on NFLG, with four samples in profile I and seven in profile II were detected and constitute two novel recombinant forms circulating in PE.

## Conclusion

Subtype B appears to be the prevalent subtype in the north-east and south-east part of Brazil followed by a high proportion of intersubtype recombinants. These results provide insights into the understanding the genesis of HIV-1 epidemic in this particular area of South America and inform vaccine design and clinical trials.

